# Detoxification of Acrylamide by Potentially Probiotic Strains of Lactic Acid Bacteria and Yeast

**DOI:** 10.3390/molecules29204922

**Published:** 2024-10-17

**Authors:** Agnieszka Maher, Karolina Miśkiewicz, Justyna Rosicka-Kaczmarek, Adriana Nowak

**Affiliations:** 1 Department of Environmental Biotechnology, Faculty of Biotechnology and Food Sciences, Lodz University of Technology, Wolczanska 171/173, 90-530 Lodz, Poland; adriana.nowak@p.lodz.pl; 2 Institute of Food Technology and Analysis, Faculty of Biotechnology and Food Sciences, Lodz University of Technology, Stefanowskiego 2/22, 90-537 Lodz, Poland; karolina.miskiewicz@p.lodz.pl (K.M.); justyna.rosicka-kaczmarek@p.lodz.pl (J.R.-K.)

**Keywords:** acrylamide, probiotics, lactic acid bacteria, yeast, detoxification, cell viability, MTT assay, bioaccumulation, DNA damage

## Abstract

Some potentially probiotic strains of lactic acid bacteria (LAB) and yeast that inhabit the digestive tract of humans are known to detoxify xenobiotics, including acrylamide (AA). The objective of the subsequent research was to evaluate the AA-detoxification capability of LAB and yeast isolated from various sources. Namely, the effect of AA was tested on the growth of LAB and yeast strains, as well in the 3-(4,5-dimethylthiazol-2-yl)-2,5-diphenyltetrazolium bromide (MTT) assay. Subsequently, the AA-binding ability of LAB and yeast was investigated in various environments, including the pH, incubation temperature, cell density, and with inanimate cells. The ability of selected LAB and yeast to reduce the genotoxicity of AA was tested on Caco-2 and Hep-G2 cell lines. The results showed that all tested strains exhibited strong resistance to AA at concentrations of 5, 10, and 50 µg/mL. Also, AA was detected in the intracellular and membrane extracts of tested strains. The most effective binding strain was *Pediococcus acidilactici* 16 at pH = 5, cell density = 10^9^ CFU/mL, and incubation temperature = 37 °C (87.6% of AA removed). Additionally, all tested strains reduced the genotoxicity of AA, with the greatest reduction observed at the highest concentration of 50 µg/mL. The phenomena of detoxification by potentially probiotic strains could reduce the toxic and harmful effects of AA exposure to humans every day.

## 1. Introduction

Acrylamide (AA), with a structure of C_3_H_5_NO, is an industrial chemical compound that is white, odorless, and very well soluble in water. AA is widely used in the industry, especially to produce polyacrylamide materials, but it is also found in heat-treated food products due to the Maillard reaction [1,2], cosmetic solutions [3], cigarette smoke [4], air pollution [5], or even environmental water from wastewater treatment processes [6]. Human exposure to AA is worldwide and inevitable. AA was identified in food for the first time by Swedish research groups in 2002, and since then, the interest in AA is thriving [1]. The International Agency for Research on Cancer (IARC) classified AA as a potentially carcinogenic substance for humans, because of its potency in inducing DNA damage and gene mutations [7]. A great number of studies proved that AA is corelated with several cancers, e.g., endometrial, ovarian, breast, renal, bladder, prostate, and colorectal [8,9,10,11,12].

As reviewed by Baskar and Aiswarya [13], AA is formed in food due to the Maillard reaction, also described as the browning reaction. This reaction occurs when amino acids (mainly asparagine) react with the α-hydroxycarbonyl groups of other compounds (for example, glucose or fructose) at high temperatures (above 120 °C) during cooking processes, like frying, baking, roasting, and toasting. The metabolism of AA in the human body is possible through two ways—enzymatically employing cytochrome P450 CYP2E1 or nonenzymatically via glutathione-S-transferase (GST). Then, in both scenarios, the reaction is conjugated with glutathione (GSH) and AA is converted to an even more reactive and genotoxic compound called glycidamide (GA) [14]. It has been proven that AA and GA are stable DNA adducts that lead to carcinogenesis [15].

Nowadays, exposure to AA toxicity involves neurotoxicity [16], genotoxicity [17], reproductive toxicity [18], hepatoxicity [19], and immunotoxicity [20]. Because of the multi-toxicity of AA, the researchers started exploring the mitigation strategies to reverse AA negative effects. Many studies proved that microorganisms can remove toxins, such as AA [21,22,23]. Here, the microorganisms of interest are potentially probiotic strains of lactic acid bacteria (LAB) and yeast. The probiotic definition from 2014 is still applicable: “live microorganisms that, when administered in adequate amounts, confer a health benefit on the host” [24]. The most studied strains are claimed to be the probiotic core group: *Bifidobacterium* (*adolescentis*, *animalis*, *bifidum*, *breve* and *longum*) and lactobacilli (*acidophilus*, *casei*, *fermentum*, *gasseri*, *johnsonii*, *paracasei*, *plantarum*, *rhamnosus* and *salivarius*) [25]. LAB species, as prokaryotes, belong to the great class of Gram-positive bacteria that can ferment sugars into lactic acid [26]. Selected strains of lactobacilli are potent in removing AA from the solution upon cell wall binding [27]. Later, Serrano-Niño et al. [28] and Zhang et al. [29] highlighted the significant role of the peptidoglycan structure in the process of toxin removal by LAB. On the other hand, commensal fungal species are also known to be the part of the human mycobiome and pose a positive health benefit to the host as probiotics [30]. The most studied and known yeast species is *Saccharomyces boulardii* (also known as *Saccharomyces cerevisiae* var. *boulardii*) [31]. Yeast, as eukaryote organisms, can also detoxify AA [32]; however, the precise mechanism has not been described yet. The studies regarding reducing AA levels in food matrixes demonstrated that yeast fermentation can minimize AA formation due to the extensive consumption of AA precursors, such as free asparagine [33].

The aim of this study was to evaluate the detoxification capability of AA by potentially probiotic strains of LAB and yeast isolated from various environments. The research involved screening these strains for their growth in the presence of AA and assessing their ability to remove AA. Additionally, the study examined the binding and bioaccumulation of AA in the strains and evaluated their potential to reduce AA-induced DNA damage in Caco-2 and Hep-G2 human cell lines.

## 2. Results

### 2.1. Lactic Acid Bacteria and Yeast Growth in the Presence of Acrylamide

**Figure 1:**
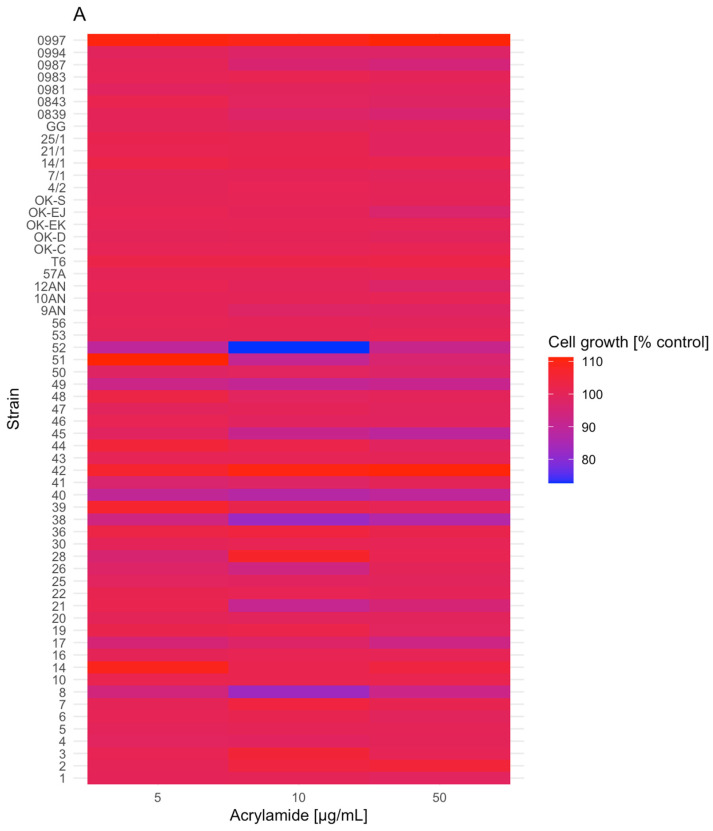

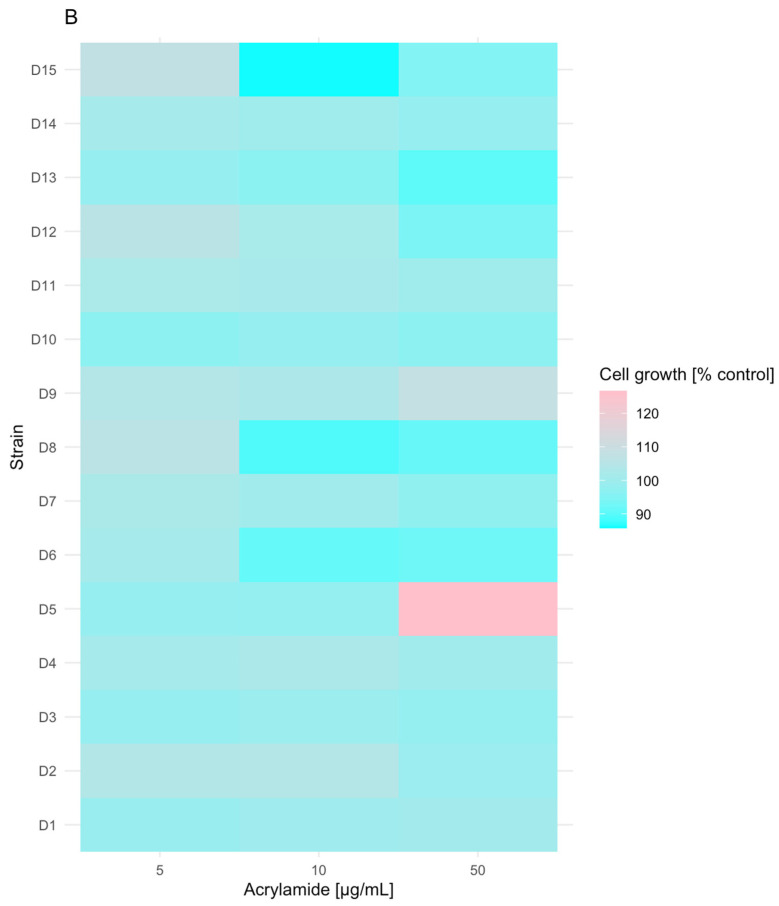
Heatmaps of lactic acid bacteria (**A**) and yeast (**B**) cell growth after acrylamide (5, 10, 50 µg/mL) exposition during 24 h incubation using the spectrophotometric method. Each data point represents the mean from eight individual wells. The evaluation was conducted in two or three independent experiments. The figures were generated using R Studio software, version 4.3.3.

The initial screening research regarding the effect of AA on the growth of 61 LAB and 15 yeast strains was evaluated using the spectrophotometric method and is presented in Figure 1A,B. However, the detailed data with calculated results (mean ± standard deviation) regarding the AA treatment on the growth of LAB and yeast strains are stored in Tables S3 and S4 included in the Supplementary Materials. The presence of AA significantly influenced the growth of the tested LAB and yeast in comparison to their negative controls, e.g., *Lactiplantibacillus plantarum* 52 (10 µg/mL), *Secundilactobacillus collinoides* 38 (50 µg/mL), *Levilactobacillus brevis* 0983 (10 µg/mL), *Lactobacillus delbrueckii* 0987 (50 µg/mL), *Kazachstani barnettii* D8 (10 µg/mL), *Kluyveromyces marxianus* D9 (50 µg/mL) (* *p* ≤ 0.0332), *S. collinoides* 38 (10 µg/mL), *Metschnikowia pulcherrima* D15 (10 µg/mL) (** *p* ≤ 0.0021), and *Wickerhamomyces anomalus* D13 (50 µg/mL) (*** *p* ≤ 0.0002).

The LAB cell viabilities ranged from 89.25 ± 1.45% (*L. plantarum* 52) to 111.27 ± 14.97% (*Lactiplantibacillus pentosus* 51) for 5 µg/mL AA, from 72.71 ± 30.86% (*L. plantarum* 52) to 110.19 ± 4.82% (*L. pentosus* 42) for 10 µg/mL AA, and from 87.02 ± 6.50% (*S. collinoides* 38) to 110.95 ± 2.52% (*Lacticaseibacillus rhamnosus* 0997) for 50 µg/mL AA. The highest LAB cell growth inhibition was observed for *L. plantarum* 52 (72.71 ± 30.86%) after exposure to 10 µg/mL AA, while the highest growth stimulation for *L. pentosus* 51 (111.27 ± 14.97%) was after exposure to 5 µg/mL AA; however, those results were not significant. Overall, the mean of the greatest LAB cell growth inhibition was observed in cells after treatment with 10 and 50 µg/mL AA, with values of 98.87 ± 6.09% and 98.95 ± 4.14%, respectively. In contrast, cells treated with 5 µg/mL AA had a cell mean calculated as 100.28 ± 3.88%.

The yeast cell growth abilities ranged from 96.80 ± 1.67% (*Hanseniaspora uvarum* D10) to 107.30 ± 6.56% (*M. pulcherrima* D15) for 5 µg/mL AA, from 85.73 ± 6.64% (*M. pulcherrima* D15) to 104.32 ± 8.49% (*Kluyveromyces lactis* D2) for 10 µg/mL AA, and from 90.26 ± 3.85% (*W. anomalus* D13) to 126.67 ± 4.31% (*Saccharomyces cerevisiae* D5) for 50 µg/mL AA. The highest yeast cell growth inhibition was observed for *M. pulcherrima* D15 (85.73 ± 6.64%) after exposure to 10 µg/mL AA, while the highest stimulation of the growth for *S. cerevisiae* D5 (126.67 ± 4.31%) was after exposure to 50 µg/mL AA; however, those results were not significant. Overall, the mean of the greatest yeast cell growth inhibition was observed in cells after treatment with 10 and 50 µg/mL AA, with values of 98.16 ± 4.39% and 99.24 ± 4.88%, respectively. In contrast, cells treated with 5 µg/mL AA displayed a cell growth mean calculated as 101.81 ± 4.37%.

**Figure 2:**
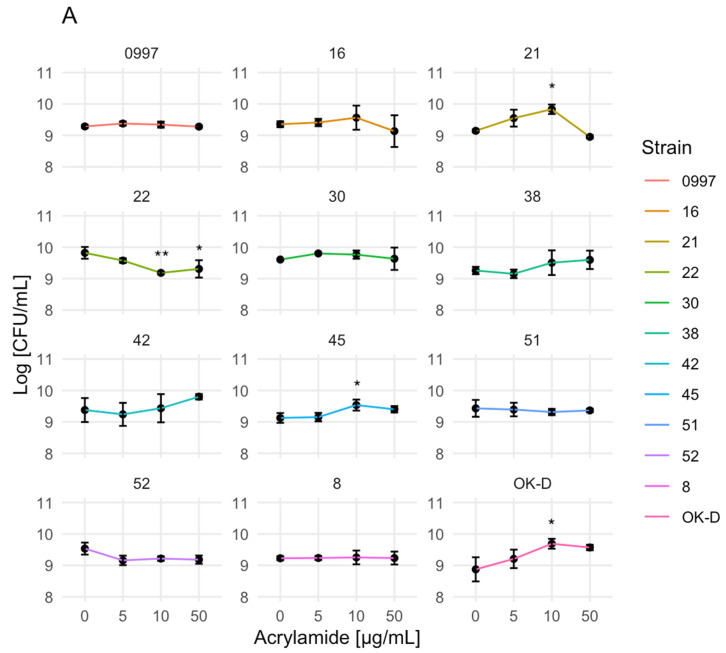

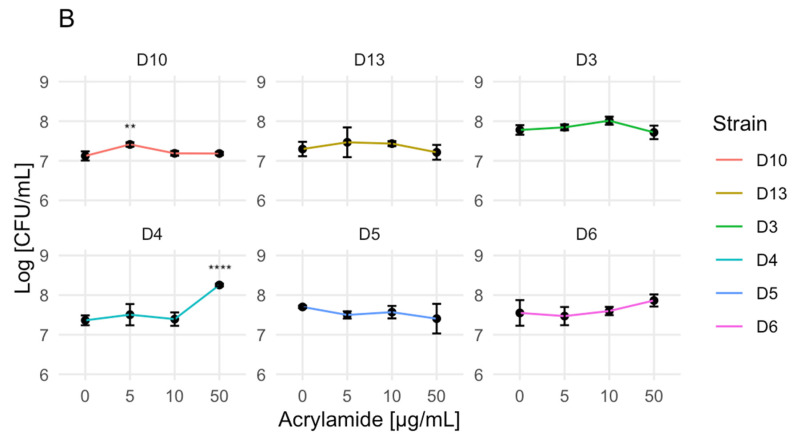
Effect of acrylamide (0, 5, 10, 50 µg/mL) on the growth of lactic acid bacteria (**A**) and yeast (**B**) strains during 24 h incubation and evaluated using the pour plate method in an appropriate agar medium. The experiment was performed with two repetitions for each strain. The evaluation was conducted with three independent experiments. Results are visualized as dots and represent the mean ± standard deviation (SD), with the significance of the difference from the unexposed control at * *p* ≤ 0.0332, ** *p* ≤ 0.0021, and **** *p* ≤ 0.0001. The figures were generated using R Studio software, version 4.3.3.

The above-mentioned utilized method provided indirect measurements that may not be correlated with viable cell counts. In addition, due to the phenomenon of an increase in cell growth using the spectrophotometric method, the effect of AA on the growth of selected LAB (Figure 2A) and yeast strains (Figure 2B) was confirmed using the pour plate method. The strains were selected based on the effect of AA on the cell yield, which included cases of increased growth, inhibited growth, or no impact. In the subsequent analysis, significant differences in growth were observed for specific strains of LAB and yeast when compared to the negative control. The presence of AA significantly influenced the growth of the tested LAB and yeast in comparison to their negative controls, e.g., *L. brevis* 45 (10 µg/mL), *Lacticaseibacillus paracasei* OK-D (10 µg/mL), *Pediococcus pentosaceus* 21 (10 µg/mL), *L. brevis* 22 (50 µg/mL) (* *p* ≤ 0.0332); *L. brevis* 22 (10 µg/mL), *H. uvarum* D10 (5 µg/mL) (** *p* ≤ 0.0021), and *S. cerevisiae* D4 (50 µg/mL) (**** *p* ≤ 0.0001). These results demonstrate that the presence of AA significantly impacted the growth of above-mentioned strains. Meanwhile, for the rest of the strains, the effect of AA on cell viabilities was not significant.

The highest LAB cell growth inhibition after AA treatment was observed for *L. brevis* 22 at a concentration of 10 µg/mL AA (9.18 ± 0.04 log CFU/mL), while the highest growth stimulation for *P. pentosaceus* 21 was at a concentration of 10 µg/mL AA (9.83 ± 0.15 log CFU/mL).

No significant inhibition of yeast cell growth was observed. However, the highest cell growth stimulation after AA treatment was observed for *S. cerevisiae* D4 (8.25 ± 0.03 log CFU/mL) after exposure to 50 µg/mL AA.

### 2.2. Lactic Acid Bacteria and Yeast Viability in the Presence of Acrylamide

**Figure 3:**
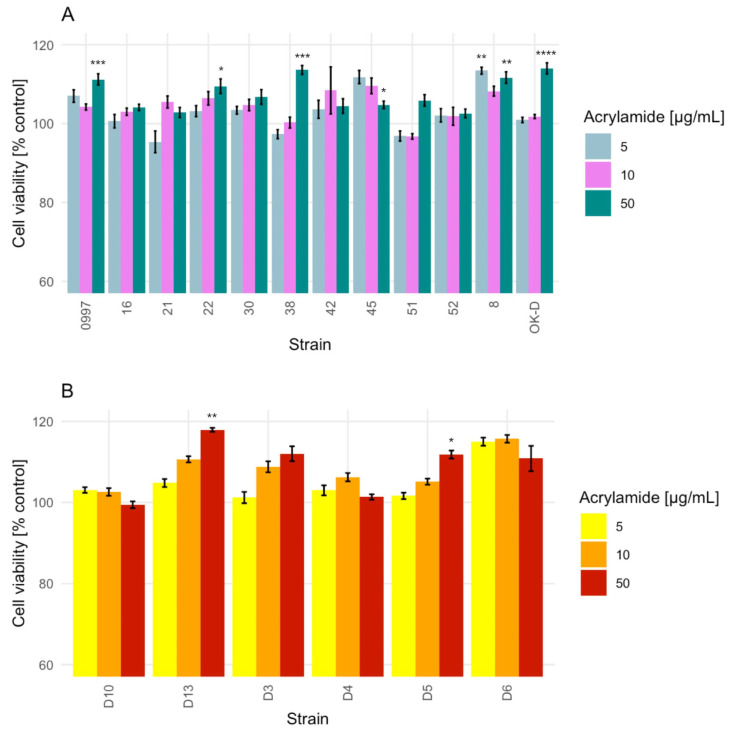
Effect of acrylamide (5, 10, 50 µg/mL) on the cell viability of lactic acid bacteria (**A**) and yeast (**B**) strains during 24 h of exposure in the MTT assay. Each data point represents the mean from eight individual wells. The evaluation was conducted using two independent experiments. Results are presented as the mean ± standard deviation (SD), with the significance of the difference from the unexposed control at * *p* ≤ 0.0332, ** *p* ≤ 0.0021, *** *p* ≤ 0.0002, and **** *p* ≤ 0.0001. The figures were generated using R Studio software, version 4.3.3.

Based on previous studies of AA impacts on cell growth, the strains of LAB and yeasts were selected for the next stage of testing cell viability with the MTT assay. The AA decreased the cell viability at a very low level (the maximum was calculated as 4.61%), or there was no decrease observed at all. The MTT assay revealed that different concentrations of AA (5 and 50 µg/mL) influenced the cell viability of some tested LAB strains (* *p* ≤ 0.0332, ** *p* ≤ 0.0021, *** *p* ≤ 0.0002, and **** *p* ≤ 0.0001), while 50 µg/mL of AA significantly differentiated the viability of a few yeast cells (* *p* ≤ 0.0332, ** *p* ≤ 0.0021) compared with their negative controls (Figure 3A,B).

In the presence of the highest AA concentration (50 µg/mL), cell viability ranged from 99.43 ± 0.84% (*H. uvarum* D10) to 117.95 ± 0.49% (*W. anomalus* D13) after 24 h of exposure. Exposure to 10 µg/mL AA resulted in cell viability from 96.76 ± 0.68% (*L. pentosus* 51) to 115.72 ± 0.95% (*Pichia fermentans* D6). Exposure to 5 µg/mL AA resulted in cell viability from 95.39 ± 2.74% (*P. pentosaceus* 21) to 115.02 ± 1.00% (*P. fermentans* D6). Among LAB strains, the greatest cell viability was observed for *L. paracasei* OK-D (114.00 ± 1.42%; 50 µg/mL AA), while the lowest was for *P. pentosaceus* 21 (95.39 ± 2.74%; 5 µg/mL AA). However, for yeast strains, the greatest cell viability was observed for *W. anomalus* D13 (117.95 ± 0.49%; 50 µg/mL AA), and the lowest was for *H. uvarum* D10 (99.43 ± 0.84%; 50 µg/mL AA).

Overall, the mean of the highest cell viability for both, LAB and yeast, was observed in cells after treatment with 10 and 50 µg/mL AA, at 105.55 ± 4.36% and 108.05 ± 5.14%, respectively. For cells treated with 5 µg/mL AA, the cell viability mean was calculated as 103.58 ± 5.36%.

### 2.3. Lactic Acid Bacteria and Yeast Binding Ability of AA

The initial studies regarding the binding assay were performed to measure the interactions between LAB or yeast and AA. In the following experiment, the ability of 37 LAB and 13 yeast strains to bind AA (5 and 50 µg/mL AA) was determined (Tables S5 and S6 in Supplementary Materials). The amount of remaining AA was from 1.93 ± 0.10 µg/mL for *P. acidilactici* 16 to 4.62 ± 0.06 µg/mL for *Lacticaseibacillus casei* 12AN (for initial 5 µg/mL AA). Meanwhile, for the higher initial AA concentration (50 µg/mL), the remaining AA was from 23.19 ± 0.28 µg/mL for *S. collinoides* 38 to 45.08 ± 0.77 µg/mL for *Pediococcus parvulus* OK-S. Based on the obtained results, the most efficient AA-binding strains of LAB and yeasts were selected for the next stages of the project, measuring the binding ability of AA by LAB and yeast under various conditions (e.g., pH, cell density, incubation time, as well as with inanimate cells, and the measurement of AA bonded in intracellular or membrane extracts).

#### 2.3.1. Effect of pH

The remaining concentration of AA varied depending on the pH and strain, with notable differences observed between strains at both 5 and 50 µg/mL AA (Table 1). At 5 µg/mL AA, *L. plantarum* 52 and *P. acidilactici* 16 showed the highest AA binding at pH 5.0 and 8.0, while *L. rhamnosus* 0997 exhibited less variation across pH values. At 50 µg/mL AA, all tested strains demonstrated relatively stable AA-binding ability across pH levels, with the most effective binding observed at pH 7.0.

#### 2.3.2. Effect of Temperature

At different incubation temperatures, it was observed that the remaining concentration of AA varied depending on the strain (Table 2). At an initial AA concentration of 5 µg/mL, *L. plantarum* 52 and *P. acidilactici* 16 showed the highest AA binding at 30 °C and 37 °C, with *L. rhamnosus* 0997 displaying relatively consistent AA binding across all temperatures (except 30 °C). At an initial AA concentration of 50 µg/mL, all strains demonstrated effective AA binding, with the most prominent binding observed at 30 °C, particularly for *L. citreum* 50 and *L. lactis* 3. Meanwhile, at a temperature of 37 °C, the strongest AA binding was marked for *K. lactis* D2. The lowest remaining AA concentrations were generally recorded at 30 °C across strains.

#### 2.3.3. Effect of Cell Density

The AA-binding ability was influenced by the concentration of microorganisms (Table 3). At 5 µg/mL AA, *L. plantarum* 52 and *P. acidilactici* 16 showed the highest AA binding at 10^9^ and 10^10^ CFU/mL, while *L. rhamnosus* 0997 displayed an increasing capability of AA binding when the microbial cell density was increasing. At 50 µg/mL AA, *L. citreum* 50 and *L. lactis* 3 showed increased AA binding with higher microbial concentrations, especially at 10^9^ and 10^10^ CFU/mL. *K. lactis* D2 demonstrated the highest AA binding at 10^10^ CFU/mL.

#### 2.3.4. Inanimate Cells

The thermally inactivated strains of LAB and yeast (T = 100 °C, 30 min) were able to bind AA at concentrations of 5 and 50 µg/mL (Table 4). The binding of AA by thermally inactivated cells was strain-dependent, with relatively similar trends observed at both 5 µg/mL and 50 µg/mL AA concentrations. At 5 µg/mL, *L. plantarum* 52 and *P. acidilactici* 16 displayed comparable AA binding, while *L. rhamnosus* 0997 had slightly lower AA binding. At 50 µg/mL, *L. lactis* 3 and *K. lactis* D2 showed the highest AA binding, exhibiting similar binding levels across strains.

When comparing inanimate cells to live cells, the remaining AA concentrations were generally lower in the live cells. The differences in AA binding between live and thermally inactivated cells were calculated by simply subtracting the following: the remaining acrylamide concentration in live cells minus the remaining concentration in inactivated cells [µg/mL]. The most significant differences in remaining AA levels were observed for the strains *P. acidilactici* 16 (0.84 µg/mL) at an initial AA concentration of 5 µg/mL and *K. lactis* D2 (7.15 µg/mL) at an initial AA concentration of 50 µg/mL. For the other tested strains, the differences in remaining AA concentrations ranged from 0.42 to 0.55 µg/mL (at 5 µg/mL initial AA) and from 1.85 to 4.57 µg/mL (at 50 µg/mL initial AA). Despite this, the reduction in AA levels by inanimate cells was comparably high (Table 4).

#### 2.3.5. Bioaccumulation of AA

AA binding occurred to a greater extent in intracellular (cell interior) than in membrane (cell wall and membrane) extracts for both AA concentrations (5 and 50 µg/mL) apart from strain *L. rhamnosus* 0997 (Table 5). At an AA concentration of 5 µg/mL, this strain bound more AA to membrane extracts (0.61 ± 0.01 µg/mL of AA remained) than to intracellular extracts (0.62 ± 0.02 µg/mL of AA remained). After incubation with a higher AA concentration (50 µg/mL), all tested strains bound AA at higher levels in intracellular extracts, with the remaining AA ranging from 0.75 ± 0.05 to 1.23 ± 0.11 µg/mL, for *K. lactis* D2 and *L. lactis* 3, respectively.

### 2.4. Lactic Acid Bacteria and Yeast Cells Decrease AA-Induced DNA Damage

**Figure 4:**
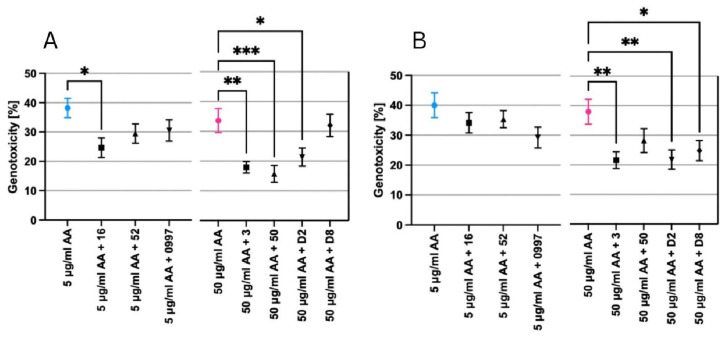
Basic endogenous DNA damage in (**A**) Caco-2 and (**B**) Hep-G2 after exposure to acrylamide in the presence of lactic acid bacteria and yeast strains, expressed as the mean percentage of DNA in the comet tail in the alkaline comet assay. Fifty cells were analyzed for each treatment. Results are presented as the mean ± standard error of the mean (S.E.M.), with the significance of the difference from the positive control at * *p* ≤ 0.0332, ** *p* ≤ 0.0021, and *** *p* ≤ 0.0002. Positive control 5 µg/mL acrylamide—blue, positive control 50 µg/mL acrylamide—pink, samples with lactic acid bacteria or yeast with addition of 5 or 50 µg/mL acrylamide—black. The figures were generated using GraphPad Prism 10 software.

In the comet assay, the effect of selected LAB and yeast strains on the reduction of DNA damage caused by AA concentrations (5 and 50 µg/mL) was evaluated (Figure 4A,B). All the tested LAB and all yeast strains demonstrated a reduction in the genotoxic effects of AA in both Caco-2 and Hep-G2 cell lines. However, only a few of LAB (strains: 16, 3, and 50) and yeast (strains: D2 and D8) significantly reduced the genotoxicity of 5 or 50 µg/mL AA compared to the positive control in the examined cell lines (* *p* ≤ 0.0332, ** *p* ≤ 0.0021, and *** *p* ≤ 0.0002).

In the Caco-2 cell line, only one strain of LAB, *P. acidilactici* 16, showed a significant reduction in DNA damage induced by 5 µg/mL AA compared to the positive control (* *p* ≤ 0.0332). Therefore, for 50 µg/mL, all tested LAB and one strain of yeast exhibited significantly different results compared to the positive control, e.g., *L. citreum* 50 (*** *p* ≤ 0.0002), *L. lactis* 3 (** *p* ≤ 0.0021), and *K. lactis* D2 (* *p* ≤ 0.0332) (Figure 4A).

**Figure 5:**
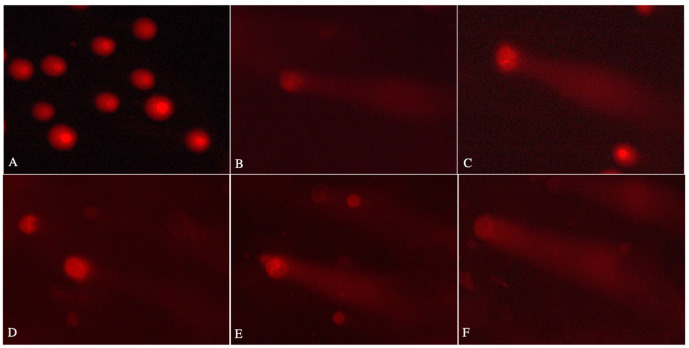
Representative images of 1 mg/mL PI-stained comets of Caco-2 cells: (**A**) untreated cells; (**B**) cells treated with 5 µg/mL acrylamide; (**C**) cells treated with 50 µg/mL acrylamide; (**D**) cells treated with 5 µg/mL acrylamide + *Pediococcus acidilactici* 16; (**E**) cells treated with 50 µg/mL acrylamide + *Lactococcus lactis* 3; (**F**) cells treated with 50 µg/mL acrylamide + *Kluyveromyces lactis* D2. Fluorescence microscopy; 200× magnification.

On the other hand, Hep-G2 cells showed higher sensitivity to the tested AA concentrations than Caco-2 cells. All tested yeast and one strain of LAB displayed a significant reduction in the genotoxicity of 50 µg/mL AA compared to the positive control (* *p* ≤ 0.0332, ** *p* ≤ 0.0021). The mean DNA damage of 50 µg/mL AA after incubation with LAB and yeast strains ranged from 21.59 ± 2.81% for *L. lactis* 3 to 35.10 ± 3.84% for *L. rhamnosus* 0997. For the lower AA concertation (5 µg/mL AA), the mean DNA damage ranged from 29.23 ± 3.30% for *L. rhamnosus* 0997 to 35.40 ± 2.84% for *L. plantarum* 52. Although a decrease in the genotoxicity of AA in the presence of LAB strains at 5 µg/mL AA was observed in Hep-G2 cells, the results were not significantly different from the positive control. Examples of comet images are presented in Figure 5.

## 3. Discussion

In the present study, the AA concentrations were selected based on a literature review and AA levels measured in foodstuffs, ranging from less than 10 to 80.92 µg/kg of food product, with the highest concentrations found in potato derivatives (potato chips, French fries) and coffee products (roasted coffee, coffee extracts) [34,35,36,37,38]. The average human intake of AA is calculated to be 0.6 µg/kg body weight/day, while in the extreme cases, consumers can be exposed to 4 µg/kg body weight/day [39,40]. For a person weighing approx. 70 kg, the average daily AA intake can be estimated as 42 µg/day. In contrast, a consumer with a diet high in AA could be exposed to 280 µg of AA daily.

The pour plate method confirmed the increased proliferation of microbial cells (similar to the 96-well spectrophotometric method) after incubation with AA. It has been proven that the influence of AA on the growth of LAB and yeast is strain- and AA dose- dependent. Certain studies have been conducted to evaluate the AA-utilization ability of microorganisms, including potentially probiotic strains of LAB or yeast. In the study of Petka et al. [41], the yeast strain of *S. cerevisiae* DSM 70478 in MRD (poor medium) was not affected by AA at any tested concentration (2.5–10 µg/mL AA) and did not utilize AA, using the nutrients in the poor medium instead. However, in the following study, *S. cerevisiae* D4 in YPG medium with the addition of 50 µg/mL showed a significant increase in cell growth compared to the negative control (**** *p* ≤ 0.0001). Additionally, the yeast strain *K. lactis* var. *lactis* DSM 70799 enhanced growth in the presence of 10 µg/mL AA, with growth calculated at 17.2 ± 8.2 × 10^5^ CFU/mL compared to the negative control at 4.8 ± 0.8 × 10^5^ CFU/mL [41]. The phenomenon of increased cell multiplication may occur because of enzymes presence in the LAB cell wall, e.g., N-acetylmuramoyl-L-alanine amidase [42] or N-acetylmuramidase [43] that are most likely responsible for AA degradation. Then, the ammonia that is released during the AA-degradation process can be metabolized and used as a carbon and nitrogen source [41,44]. However, the study concerning the direct deamination process of AA by LAB or yeast cells has not been described yet. In a subsequent study, the 1000 µg/mL of AA was tested for 24 h using the pour plate method on two strains: *K. lactis* D2 and *L. rhamnosus* 0997. Despite the high AA concentration, no negative impact on the microbial cells was observed. The cell growth values achieved were 9.57 × 10^6^ CFU/mL (negative control—5.40 × 10^6^ CFU/mL) and 1.03 × 10^9^ CFU/mL (negative control 3.40 × 10^9^ CFU/mL) for *K. lactis* D2 and *L. rhamnosus* 0997, respectively. Petka et al. [44] also conducted the experiment with high doses of AA (1000 µg/mL of AA) and observed intense cell growth (described as +++) in most of the *Lactobacillus* strains in MRD medium compared to those treated with lower doses of AA (0–500 µg/mL of AA), which were described as good growth (++).

A number of studies have been performed regarding the cytotoxicity of AA in cell lines, showing that AA decreases cell growth, increases ROS (reactive oxygen species) production, and attenuates mitochondrial functions [45,46,47]. In the study of Li et al. [48], the reduced cell growth of the human gastric epithelial cell line (GES-1) and Caco-2 cells was observed after exposure to AA, with IC_50_ (half-maximal inhibitory concentration) values reaching 3.48 and 4.66 mM, respectively. The detoxification of AA was also evaluated by Fan et al. [49] who observed that the heat-processing of quercetin and myricetin can alleviate the AA-induced cytotoxicity in rat small intestine (IEC-6) cells. However, the research about AA decreasing the cell viability of LAB or yeast cells is still lacking. In the study of Kovár et al. [50], the cell growth of *Schizosaccharomyces pombe* after AA treatment with lower doses (1 mM) showed an increase in cellular metabolism to balance the internal homeostasis. However, the higher AA concentrations (10–40 mM) resulted in a decrease in metabolic activity. In accordance with the following study, for the highest tested AA concentration—50 µg/mL—a decrease in cell viability was observed in only three out of the six yeast strains tested when compared to the AA-unexposed negative control. Moreover, significant differences from the negative controls were found in only two yeast strains: *S. cerevisiae* D5 (* *p* ≤ 0.0332) and *W. anomalus* D13 (** *p* ≤ 0.0021). For both strains, cell proliferation was significantly increased compared to the negative control. Previously, the MTT assay was primarily used in the mammalian cell studies [51]; however, it can be applied in microbiological studies for eucaryotic (such as yeast) and prokaryotic (such as LAB) cells as well [52]. In mammalian cells, which are eukaryotic, the MTT assay measures cell viability by assessing mitochondrial activity, as the dye is reduced in the mitochondria of metabolically active cells, producing a color change [53]. Similarly, in yeast, which are also eukaryotic, the MTT assay operates through mitochondrial reduction. However, in prokaryotic bacteria, which lack mitochondria, the reduction of MTT to formazan is carried out by bacterial dehydrogenases [54]. In our study, during MTT testing, AA did not induce a high decrease in the cell viabilities of tested strains. One of the LAB strains—*P. pentosaceus* 21—exhibited the highest decrease in cell viability calculated as 4.61% after exposure to 5 µg/mL AA. This could indicate that 5 µg/mL AA induced some stressful effects and cells had not yet activated their defense mechanisms. However, at higher AA concentrations, the decrease in cell viability of *P. pentosaceus* 21 was reversed and cell growth was slightly increased up to 105.47% and 102.82%, for 10 and 50 µg/mL AA, respectively. Hypothetically, an increase in cell growth at higher AA concentrations might occur because of the cellular stress response, which can activate the defense mechanisms in microbial cells. As mentioned previously, the increased cell viability may take place due to AA degradation by microorganisms and then later utilized as a source of carbon and nitrogen. It was confirmed that the effect of AA on the cell viability of microbial cells depends on the strain and AA concentration.

Recently, Lv et al. [55] conducted detailed research about the mechanisms of AA binding by *L. plantarum* ATCC8014 peptidoglycan. It was proven that AA adsorption requires physisorption and chemisorption processes. Therefore, those results indicate that carboxyl, amino, and hydroxyl functional groups serve as the primary binding sites for AA in peptidoglycan. The study also revealed the involvement of hydrogen bonds, hydrophobic forces, and amidases in the adsorption process [55]. Accordingly with the study by Zhang et al. [29], the peptidoglycan from *L. plantarum* 1.0065 had the highest ability out of the four tested strains to bind AA with 87%. Importantly, alanine in the peptidoglycan was positively correlated with a significant impact on AA binding. Later, Zhang et al. [56] also investigated the AA removal by some *Lactobacillus* strains (2 µg/mL AA) and the greatest AA reduction was observed for strains of *L. plantarum* 1611 (22.53%) and *L. pentosus* ML32 (18.91%). However, the above-mentioned researchers evaluated AA removal, as well with the addition of 0.1–2% (*m*/*v*) bovine serum albumin (BSA), and then, the reduction rates achieved even higher values, especially for 0.5% BSA added to bacterial cells of *L. pentosus* ML32 (35.94% of AA removed). Albedwawi et al. [57], utilizing a Box-Behnken design, demonstrated that within an 18 h incubation period, *B. breve* achieved optimal AA reduction, by removing over 64% under a pH of 4.5–5.0 and a temperature of 32 °C. In contrast, *L. plantarum* was able to remove up to 35% of AA at pH 4.5 and a temperature of 32–38 °C and exhibited the greatest AA reduction at a temperature of 34–42 °C within 14 h. However, the optimal AA removal was demonstrated at a temperature of 37 °C, pH 4.5–5.0, for 14–15 h, displaying a 60% AA reduction for *L. plantarum*. Meanwhile, compared to the results of the following paper—for strain *L. plantarum* 52—the greatest AA reduction was measured at pH = 5 for a 24 h incubation period at 37 °C (86.6% of AA removed). Simultaneously, a high binding capacity was also measured at pH = 8 for *L. plantarum* 52 (80.2% of AA removed). Studies indicated that during the production of fermented foods, where the pH is lower, AA levels significantly decrease [44,58]. For instance, during bread baking, fermentation with LAB reduces AA levels due to sugar consumption by LAB and acid production, which inhibits the Maillard reaction and subsequently reduces AA formation in baked bread [59]. On the other hand, the primary mechanism by which yeasts remove AA is through the metabolism of its precursors, such as asparagine. To date, there are no known reports of yeasts incorporating AA into their cell envelope or hydrolyzing AA via acrylamidase production [57]. However, there are several studies about mycotoxin binding by yeast. Research by Piotrowska and Masek [60] and Bzducha-Wróbel [61] found that β-d-glucans from yeast cell walls are responsible for toxin binding. Recently, due to the limitation of utilizing live cells, the microbial adsorbents containing β-d-glucans from the yeast cell wall are being extracted and utilized in decontamination studies.

In our study, it was clearly indicated that the AA binding was found to be strain- and AA-concentration-specific. Each strain has different cell wall components, also leading to varying numbers of binding sites and capacities in binding certain amounts of toxins [28,44]. The phenomenon of the higher effectiveness of cell binding at higher cell densities (10^9^ and 10^10^ CFU/mL) can be explained by the greater number of cells providing more available binding sites, resulting in a stronger affinity to bind and reduce xenobiotics, including AA [62,63]. On the other hand, some researchers have concluded that a microorganism’s ability to bind toxins is linear, with cells possessing undefined binding sites [64]. Secondly, the pH value also signifies the binding capacity. Several studies have explored how pH influences the binding of toxins (including AA) by LAB [27,29,63]. Choi et al. [63] demonstrated that at a low pH (pH = 2.0–3.0), the AA reduction was minimal. This was explained by the competition between AA and protons for binding to negatively charged cell wall components (e.g., teichoic acid) at a low pH, which results in less AA binding [63]. In contrast, Ma et al. [65] reported that acid treatment of LAB increases the hydrophobicity via the denaturation of cell surface proteins, resulting in a greater number of available binding sites for toxins. Nevertheless, the effects of pH on cell-wall-binding properties remain largely unexplored. We suggest that microbial cells at an extremely low or high pH may behave similarly to inanimate cells (discussed later in this section). However, further research is needed to validate this hypothesis. Therefore, there are some studies focusing on the inanimate cell and their ability to bind toxins. For instance, Piotrowska [66] studied ochratoxin A adsorption by dead cells of *Lactobacillus* spp., and the results were correspondent with those obtained here. Namely, the xenobiotic adsorption by dead cells is possible and may proceed due to increased permeability of the external layers of the cell wall as the results of protein denaturation caused by treatment at high temperature. Also, after heat treatment, the cell wall properties change from hydrophilic to hydrophobic. In contrast, cells that have not been exposed to high temperatures can bind toxins in the presence of hydrophobic pockets on the cell wall surface [67]. In accordance with our study, we can assume that the incubation temperature also contributes to the properties of the cell wall. Probably, the most effective binding likely occurs at the optimal incubation temperature, where the highest number of hydrophobic pockets are present. For all the tested strains, this optimal temperature was found to be 30 or 37 °C depending on the strain. Based on the results, the AA-binding character suggests that the mechanism of binding by LAB is passive—occurs depending on the hydrophobic and electrostatic interactions between cells and toxins [68]. These findings indicate that inanimate cells are a promising tool in detoxification processes and can be used as postbiotics.

To the best of the authors’ knowledge, there is no existing literature on AA bioaccumulation in intracellular or membrane extracts of bacterial and yeast cells. In the study by Leska et al. [69], pesticides were found to bind to the intracellular and membrane extracts of LAB strains, with the highest concentration detected in intracellular extracts. The most significant concentration was observed for chlorpyrifos (21.27 ± 0.39 μg/mL where the initial concentration was 100 μg/mL) in *P. parvulus* OK-S. Additionally, Hu et al. [70] reported the intracellular bioaccumulation of uranium in strains of *Stenotrophomonas* sp. Heavy metals, like cadmium, also show strong potential for intracellular bioaccumulation in LAB. Microscopic observations by Gerbino et al. [71] revealed a cadmium presence within the intracellular parts of *Lactobacillus kefir* JCM 5818. Another study utilized LAB strains for the bioremediation of water contaminated with heavy metals, where *L. brevis* 20 accumulated nickel at the highest rate of 82% [72]. *Lactobacillus* strains have also been employed in bioremediation processes for lead and cadmium [73]. In our study, it has been shown that AA is accumulated in LAB and yeast extracts, rather at low levels. The highest bioaccumulation was observed for an initial 5 μg/mL of AA—0.62 ± 0.02 μg/mL (12.40% of AA accumulated)—while for an initial 50 μg/mL of AA, it was 1.51 ± 0.04 μg/mL (3.02% of AA accumulated). On the other hand, beneficial minerals for human health, such as selenium, were also found to bioaccumulate in the intracellular extracts of LAB and yeast [74,75]. This phenomenon may be advantageous for introducing selenium deficiencies in the daily diet by producing selenium-enriched functional foods.

Since the results regarding AA binding in our study are very promising, in humans, the binding of AA (and other toxic compounds) to the cell surface or its absorption into the interior of the microorganism is a recognized detoxification method. As these microorganisms pass through the digestive tract and are eliminated with feces, they carry the absorbed toxic compounds, including AA. This process reduces the contact between carcinogenic compounds and the intestinal epithelium, thereby minimizing their negative impact on the body [76,77].

To the best of the authors’ knowledge, no study has been published on the reduction of induced DNA damage caused by AA in Caco-2 or Hep-G2 cell lines utilizing LAB or yeast. However, Zhao et al. [78] have examined the protective effect of *L. plantarum* ATCC8014 on AA-induced oxidative damage in rats, and 1 × 10^9^ CFU/mL of the chosen strain could effectively reduce the injury of AA to rats’ bodies. Another LAB strain, *Lactobacillus reuteri* JCM 1112, was observed to ameliorate the chronic AA-induced glucose metabolism disorder through the bile acid–TGR5–GLP-1 axis and modulate intestinal oxidative stress in mice [79]. In the study of Cuevas-González et al. [80], the strains *L. fermentum* J10, *L. pentosus* J24, J26, and J27 were found to protect human erythrocytes from AA-induced oxidative damage. In addition, *Lactobacillus* GBE17 and GBE29 were potent in protecting Caco-2 cells from generating ROS after AA exposition [81]. In the study of Zamani et al. [82], L-carnitine (100 and 200 µM) was observed to effectively prevent AA genotoxicity by alleviating oxidative stress in human lymphocytes, leading to a reduction in micronuclei frequencies. Moreover, it was discovered that hydroxytyrosol is the substance with potency in reducing AA-induced genotoxicity, as well as decreasing intracellular ROS formation and mitigating GSH depletion caused by AA. Additionally, hydroxytyrosol was found to enhance the expression of γ-GCS in HepG2 cells treated with 10 mM AA [83]. In our study, *L. citreum* 50 decreased the AA-induced genotoxicity at the greatest level—15.71 ± 2.81% (the positive control—33.60 ± 4.03%) in the Caco-2 cell line.

In the future research, it would be recommended to focus on AA removal under in vitro gastrointestinal conditions (e.g., INFOGEST system) or in a dynamic artificial gastrointestinal tract (e.g., SHIME), as well as exploring the molecular mechanisms responsible for the obtained outcome. In the above conducted study, it was shown that tested LAB and yeast strains are promising tools in mitigating the harmful effects of environmental and diet toxins, such as AA.

## 4. Materials and Methods

### 4.1. Culture Vessels, Chemicals, and Other Materials

Acrylamide (AA) for stock solution preparation, propidium iodide (PI), dimethyl sulfoxide (DMSO), MTT, ethylenediaminetetraacetic acid (EDTA), DeMan, Rogosa, and Sharpe (MRS) Broth and agar, sodium chloride (NaCl), sodium hydroxide (NaOH), hydrochloric acid (HCl), phosphate buffered saline (PBS), high-glucose Dulbecco’s Modified Eagle Medium (DMEM), trypsin, 4-(2-hydroxyethyl)-1-piperazineethanesulphonic acid (HEPES), the streptomycin–penicillin mixture for cell cultures, trypan blue, low-melting-point (LMP) agarose, normal-melting-point (NMP) agarose, Tris, and Triton X-100 were purchased from Merck Life Science (Warsaw, Poland). YPG Broth and agar was supplied from BTL (Lodz, Poland). Fetal bovine serum (FBS), GlutaMAX^TM^, and TrypLE^TM^ Express were purchased from Thermo Fisher Scientific (Waltham, MA, USA). The human cell lines colon adenocarcinoma Caco-2 and hepatocellular carcinoma cell line Hep-G2 were purchased from the CLS Cell Line Service GmbH (Eppelheim, Germany) and DSMZ German Collection of Microorganisms and Cell Cultures GmbH (Leibniz, Germany). For AA analysis via LC-MS/MS, acrylamide (99%) was procured from Merck Life Science (Warsaw, Poland), and 2,3,3-d3-acrylamide (>98%) was obtained from Cambridge Isotope Laboratories, Inc. (Andover, MA, USA); acetonitrile (99.9%) was purchased from Merck Life Science (Warsaw, Poland); formic acid, methanol, and Carrez reagents were supplied by POCH S. A. (Gliwice, Poland).

### 4.2. Strains and Growth Conditions

A total number of 61 LAB and 15 strains of yeast were used for this research. Most of the LAB strains (*Levilactobacillus brevis*, *Lactococcus lactis*, *Lactiplantibacillus plantarum*, *Pediococcus acidilactici*, *Leuconostoc mesenteroides*, *Pediococcus pentosaceus*, *Limosilactobacillus fermentum*, *Secundilactobacillus collinoides*, *Leuconostoc citreum*, and *Lactococcus raffinolactis*), were isolated from sourdough. Several LAB strains were isolated from sour cucumbers: *Lentilactobacillus diolivorans*, *L. brevis*, *Lacticaseibacillus paracasei*, *L. plantarum*, and *Pediococcus parvulus.* The other LAB strains were isolated from various sources, e.g., sauerkraut, curdled cow milk, chicken pile with litter, infant/ human feces, flowers of *Papaver rhoeas*, flowers of *Lavandula augustifolia*, fresh fermented honey from the beekeeper, nectar-heather honey, and Jerusalem artichoke, and strains were as follows: *Ligilactobacillus salivarius*, *Loigolactobacillus coryniformis*, *L. casei*, *P. acidilactici*, *P. pentosaceus*, *L. plantarum*, *P. acidilactici*, and *Lentilactobacillus farraginis.* Additionally, a commercial probiotic *Lacticaseibacillus rhamnosus* GG (human feces) was evaluated.

Yeast strains were isolated only from food products, e.g., cider, rye/wheat flour, sourdough, sour cucumber, sour beet root, wild grape, and multivitamin drink, as well as blackberry fruit, and strains were as follows: *Saccharomyces cerevisiae*, *Pichia fermentans*, *Wickerhamomyces anomalus*, *Kazachstania barnettii*, and *Hanseniaspora uvarum*. Additionally, a commercial strain, *S. cerevisiae* from BIO STAR company, was also acquired (in the study defined as strain D5).

All above-mentioned, non-commercial strains were acquired from their own collection of the Department of Environmental Biotechnology, Lodz University of Technology. The isolated strains from various sources were identified either via API tests or by means of the MALDI-TOF MS technique.

Furthermore, LAB (*Lactobacillus acidophilus* ŁOCK 0839, *Amylolactobacillus amylophilus* ŁOCK 0843, *L. plantarum* ŁOCK 0981, *L. brevis* ŁOCK 0983, *Lactobacillus delbrueckii* ŁOCK 0987, *L. mesenteroides* ŁOCK 0994, *L. rhamnosus* ŁOCK 0997) and yeast strains (*Kluyveromyces lactis* ŁOCK 0028 D2, *Kluyveromyces marxianus* ŁOCK 0024 D9) were applied from the Pure Culture Collection of the Institute of Fermentation Technology and Microbiology (ŁOCK), Lodz University of Technology. Additionally, *Metschnikowia pulcherrima* NCYC 747 D3 from the National Collection of Yeast Cultures (Norwich, UK) and *M. pulcherrima* CCY-2-145 D15 from the Culture Collection of Yeast (Bratislava, Slovakia) were tested. All strains used in the study with a described isolation source are listed in the Supplementary Materials (Tables S1 and S2).

Some LAB strains have been evaluated for various probiotic properties, such as antagonistic activity against pathogens, antibiotic susceptibility, survival in a simulated gastrointestinal tract, and adhesion capabilities. These studies were conducted under in vitro conditions and have been published [69,84,85,86]. However, since these strains have not yet been tested in vivo, they are referred to as “potentially” probiotic in the paper.

Isolated LAB and yeast were stored in Cryobanks™ (Murrieta, CA, USA) at −20 °C. Before the experiments, the strains were activated, passaged three times (3% inoculum), and cultured in MRS/YPG broth for 24 h at 30/37 °C.

### 4.3. AA Stock Preparation and Storage

AA stocks with a concentration of 1 mg/mL were prepared in the appropriate MRS/YPG medium or HPLC-grade water. Stocks were stored at 4 °C.

### 4.4. Effect of AA on the Growth of Strains

#### 4.4.1. Spectrophotometric Method

LAB and yeast were activated and plated (3% inoculum) in a 96-well microplate. The final concentrations of AA tested were 5, 10, and 50 µg/mL. Negative controls were unexposed bacterial and yeast cells in MRS/ YPG broth. The plates were incubated at 30/37 °C for 24 h, and then, the absorbance (600 nm) was measured in a microplate reader (TriStar^2^ LB 942, Berthold Technologies GmbH & Co. KG, Bad Wildbad, Germany). The absorbance of the control sample, representing untreated cells, was considered to reflect 100% cell viability. Cell viability was calculated according to the following formula [%]: average absorbance of the actual sample × 100/average absorbance of the control.

#### 4.4.2. Pour Plate Method

Activated LAB and yeast samples were prepared, consisting of 3% inoculum and appropriate concentrations of AA (5, 10, and 50 µg/mL), in MRS/ YPG medium. A suitably diluted AA stock with a concentration of 1 mg/mL was used to prepare the samples. The samples were incubated at 30/37 °C for 24 h. Then, 10-fold dilutions were made in 0.85% physiological salt for each strain with selected AA concentrations, and they were sown on Petri plates and covered with the appropriate medium—MRS/ YPG with agar. The seeded plates were incubated at 30/37 °C for 48 h, and then, the grown colonies were counted.

### 4.5. MTT Assay

The MTT assay was utilized to evaluate cell viability of LAB and yeast. The protocol was performed according to Hegyi et al. [54] with modifications. Activated LAB and yeast were plated in a 96-well microplate. The final concentrations of AA tested were 5, 10, and 50 µg/mL. Negative controls were unexposed bacterial and yeast cells in MRS/ YPG broth. The plates were incubated at 30/37 °C for 24 h and then centrifuged (4000 rpm, 10 min), and the supernatants were removed; 100 µL of PBS was added to each well and mixed thoroughly. Then, cells in PBS were transferred to a new 96-well plate with PBS (1:9, *v/v*; 10× dilution). The next step was to add MTT stock solution to each well (MTT final concentration, 0.8 mg/mL). Incubation was carried out at 30/37 °C for 2 h. After incubation, the plates were centrifuged (4000 rpm, 10 min), and the supernatants were removed. Next, DMSO was added to each well and shaken vigorously on a shaker (10 min, room temperature) to thoroughly dissolve the formed formazan. After 10 min, the absorbance was measured in a microplate reader at a wavelength of 595 nm (using a 620 nm reference filter) in a microplate reader (TriStar^2^ LB 942, Berthold Technologies GmbH & Co. KG, Bad Wildbad, Germany). The absorbance of the control sample, representing untreated cells, was considered to reflect 100% cell viability. Cell viability [%] was determined using the following formula: (absorbance of the sample/absorbance of the control) × 100%.

### 4.6. AA-Binding Assay

#### 4.6.1. Whole Live LAB and Yeast Cells

Overnight LAB and yeast cultures were centrifuged, and the biomass was washed with HPLC-grade water (the operation was repeated two or three times until the culture medium was completely washed out). The biomass was suspended in HPLC-grade water with the appropriate AA concentration (5 or 50 µg/mL), in 5 pH options (4.0; 5.0; 6.0; 7.0; 8.0) ± 0.1. Cell density was determined using a densitometer (DEN-1, Grant-bio, Cambridge, UK). For this purpose, the samples were diluted to obtain densities of 10^6^ and 10^8^ CFU/mL. To obtain a cell density of 10^10^ CFU/mL, the samples were concentrated. Incubation was carried out at 4 °C, 20 °C, and 37 °C on a shaker. After incubation, the samples were centrifuged, 1.5 mL of supernatants were taken into an Eppendorf, 0.2 mL of Carrez I and II reagents were added to deproteinize the samples, and then, the samples were centrifuged again. The supernatants were then filtered (0.22 μm) and frozen at −20 °C until LC-MS/MS analysis.

#### 4.6.2. Thermally Inactivated LAB and Yeast Cells

The above procedure (Section 4.6.1.) was also executed for heat-inactivated LAB and yeast strains (100 °C, 30 min).

#### 4.6.3. Intracellular Extracts (ICEs) and Membrane Extracts (MEs)

To determine the mechanisms of AA binding, intracellularly (detection in intracellular extracts—ICEs) and to cell membranes (detection in membrane extracts—MEs), the samples from whole live cells were subjected to sonification on ice (5 min, amplitude 50, pulse 6 s, pause 2 s; Hielscher Ultrasonics GmbH homogenizer, Teltow, Germany). The supernatants were then subjected to deproteinization with Carrez I and II reagents, filtered (0.22 μm), and frozen at −20 °C until LC-MS/MS analysis.

### 4.7. Determination of AA Content Using the LC-MS/MS Method

The determination of AA content was performed using an LC-MS/MS system consisting of a Transcend TLX-1 liquid chromatograph connected to a Q-Exactive hybrid mass spectrometer from Thermo Scientific equipped with an ESI ionization source and Aria 1.6.3, Thermo Xcalibur 2.2 software and Qexactive Tune 2.1. The chromatographic analysis conditions were as follows: column: Accucore™ C18 (Sigma-Aldrich (St. Louis, MO, USA)), 2.6 μm, 100 mm × 3.0 mm i.d.; eluents: aqueous solution of 0.1% formic acid as mobile phase A and a mixture of methanol/formic acid (99.9:0.1; *v/v*) as mobile phase B; flow: 0.3 mL/min; column temperature: 40 °C. The MS/MS parameters were as follows: capillary voltage—3500 V; gas drying temperature—400 °C; gas flow (N_2_)—8 l/min, collision energy—25 kV. The instrument was used in positive ion scanning mode in the *m*/*z* range from 50 to 1000. After ESI ionization, the parent and fragment ions of AA were monitored at *m*/*z* 72 and 55. AA was identified based on the retention time and mass spectrum in comparison to the reference AA.

### 4.8. Cell Line Cultures

Caco-2 and Hep-G2 human cell lines were cultured in high-glucose DMEM and Ham’s F12 medium, respectively. Simultaneously, the media were additionally supplemented either with 5 or 10% FBS, 2 or 4 mM GlutaMAX^TM^, 25 mM HEPES or 0.1 U mL^−1^ insulin, 100 µg/mL streptomycin, and 100 IU/mL penicillin. The cells were incubated in a humidifier (37 °C, 5% CO_2_) for 7–10 days to achieve approx. 80% confluence. Every 3 days, the cells were washed using 0.1 M PBS (pH 7.2) and the cell culture medium was changed. When the cells achieved confluence, they were detached from the culture using TrypLE^TM^ Express (37 °C, 10 min) following the manufacturer’s instructions. Sterile PBS was added to the detached cells, and the cell suspension was removed from the culture flask. The cells were then centrifuged (307× *g*, 5 min) and cell pellets were re-suspended in fresh culture medium. Cell counting was performed using a hemocytometer, and viability was assessed via trypan blue exclusion.

### 4.9. Single-Cell Gel Electrophoresis Assay (Comet Assay)

The comet assay was conducted to assess the genotoxicity of AA in the presence of LAB and yeast strains using Caco-2 and Hep-G2 cell lines. Eppendorf tubes were prepared with 1 × 10^5^ cells per sample in 1 mL of appropriate culture medium without supplements. The final concentration of all test samples was set to 20% (*v/v*). Control samples contained cells not exposed to any strains or AA, while positive control samples consisted of cells exposed only to AA. All samples were incubated (60 min, 37 °C), followed by centrifugation (15 min, 4 °C, 182× *g*). The supernatant was decanted, and low-melting-point (LMP) agarose was added to the cell pellet at 37 °C. The suspension was then applied onto pre-warmed NMP double-layered slides and covered with coverslips. The samples were placed on a chilling plate to solidify the agarose. Next, alkaline lysis was performed by incubating the slides in a lysis buffer (2.5 M NaCl, 1% Triton X-100, 100 mM EDTA, 10 mM Tris, pH 10) for 60 min at 4 °C. Following lysis, the buffer was removed, and the slides were flooded with unwinding buffer (300 mM NaOH, 1 mM EDTA) for 20 min at 4 °C. The slides were then transferred to an electrophoresis apparatus, where electrophoresis was carried out in an electrophoretic buffer (300 mM NaOH, 1 mM EDTA, pH > 13) for 20 min at 21 V and 29 mA. After electrophoresis, the slides were neutralized, dried, and stained with PI (1 µg/mL) for 60 min at 4 °C. Comet analysis was performed using a fluorescence microscope (Nikon) at a 200 × magnification, equipped with a Nikon Digital Sight DS-U3 camera and Lucia Comet v.7.0 software (Laboratory Imaging, Prague, Czech Republic). In each trial, 50 randomly selected comets were analyzed to determine the percentage of DNA in the comet tail. Results are expressed as the mean ± S.E.M.

### 4.10. Statistical Analysis

The data were analyzed using one-way analysis of variance (ANOVA), followed by Dunnett’s multiple-comparisons test (GraphPad Prism 10, Boston, MA, USA). Significant differences were accepted at * *p* ≤ 0.0332, ** *p* ≤ 0.0021, *** *p* ≤ 0.0002, and **** *p* ≤ 0.0001. The results were presented either as the mean ± standard deviation (SD) or standard error of the mean (S.E.M.).

## 5. Conclusions

It can be concluded that AA at different concentrations influences the microbial cell growth differently. This phenomenon is AA-dose-dependent and strictly dependent on strain specificity. The performed study proved that some of strains exhibit an increase in cell growth after exposure to AA due to possible defense mechanisms or metabolic adaptations. The binding assay revealed that LAB and yeast can effectively bind AA, with efficiency influenced by environmental conditions, such as the pH and temperature, and strain characteristics. Generally, the most effective binding occurred at pH 5 and 8. LAB and yeast strains detoxify AA the most at cell densities of 10^9^ and 10^10^ CFU/mL.

These results suggest the huge potential of LAB and yeast application in detoxifying AA-contaminated environments and as additives in the human diet. Research on this topic needs to be continued.

## Figures and Tables

**Table 1:** The ability of LAB and yeast cells to bind AA to the cell wall after 24 h of incubation at 37 °C at different pH values. The cell density was 10^9^ CFU/mL. Results are presented as the mean from two measurements ± standard deviation (SD).

Initial Concentration of AA 5 µg/mL					
Strain	Remaining Concentration of AA [µg/mL]				
	pH 4.0	pH 5.0	pH 6.0	pH 7.0	pH 8.0
*L. plantarum* 52	2.66 ± 0.12	0.67 ± 0.01	1.31 ± 0.05	2.23 ± 0.01	0.99 ± 0.10
*P. acidilactici* 16	2.64 ± 0.10	0.62 ± 0.04	1.08 ± 0.03	1.93 ± 0.08	0.72 ± 0.05
*L. rhamnosus* 0997	2.71 ± 0.11	2.49 ± 0.18	2.45 ± 0.18	2.47 ± 0.13	1.45 ± 0.07
**Initial concentration of AA 50 µg/mL**					
**Strain**	**Remaining concentration of AA [µg/mL]**				
	**pH 4.0**	**pH 5.0**	**pH 6.0**	**pH 7.0**	**pH 8.0**
*L. citreum* 50	28.73 ± 0.21	29.02 ± 0.44	29.88 ± 0.57	23.54 ± 0.43	32.00 ± 0.33
*L. lactis* 3	28.42 ± 0.31	26.68 ± 0.38	29.99 ± 0.42	23.62 ± 0.56	32.13 ± 0.49
*K. lactis* D2	29.48 ± 0.34	27.34 ± 0.24	29.57 ± 0.31	20.24 ± 0.60	29.59 ± 0.36
*K. barnettii* D8	27.83 ± 0.52	26.84 ± 0.35	30.89 ± 0.32	26.38 ± 0.53	30.07 ± 0.21

**Table 2:** The ability of LAB and yeast to bind AA to the cell wall after 24 h of incubation at different temperatures: 4 °C, 20 °C, 30 °C, and 37 °C. The pH was 7.0 and the cell density was 10^9^ CFU/mL. Results are presented as mean from two measurements ± standard deviation (SD).

Initial Concentration of AA 5 µg/mL				
Strain	Remaining Concentration of AA [µg/mL]			
	T = 4 °C	T = 20 °C	T = 30 °C	T = 37 °C
*L. plantarum* 52	2.65 ± 0.03	2.96 ± 0.05	1.80 ± 0.02	2.23 ± 0.03
*P. acidilactici* 16	2.80 ± 0.10	2.91 ± 0.12	2.11 ± 0.00	1.93 ± 0.10
*L. rhamnosus* 0997	2.80 ± 0.07	2.83 ± 0.04	2.12 ± 0.01	2.47 ± 0.04
**Initial concentration of AA 50 µg/mL**				
**Strain**	**Remaining concentration of AA [µg/mL]**			
	**T = 4 °C**	**T = 20 °C**	**T = 30 °C**	**T = 37 °C**
*L. citreum* 50	27.73 ± 0.54	29.97 ± 0.37	21.13 ± 0.43	23.54 ± 0.39
*L. lactis* 3	28.01 ± 0.66	29.42 ± 0.39	22.20 ± 0.56	23.62 ± 0.21
*K. lactis* D2	27.85 ± 0.43	28.63 ± 0.44	23.49 ± 0.21	20.24 ± 0.15
*K. barnettii* D8	29.25 ± 0.31	29.19 ± 0.22	22.68 ± 0.35	26.38 ± 0.41

**Table 3:** The ability of LAB and yeast to bind AA to the cell wall after 24 h of incubation at 37 °C at various cell densities: 10^6^, 10^8^, 10^9^, 10^10^ CFU/mL. The pH was 7.0. Results are presented as the mean from two measurements ± standard deviation (SD).

Initial Concentration of AA 5 µg/mL				
Strain	Remaining Concentration of AA [µg/mL] (AA Bound per 1 Cell)			
	10^6^	10^8^	10^9^	10^10^
*L. plantarum* 52	3.05 ± 0.12 (1.95 × 10^−6^)	3.19 ± 0.03 (1.81 × 10^−8^)	2.23 ± 0.05 (2.77 × 10^−9^)	2.63 ± 0.12 (2.37 × 10^−10^)
*P. acidilactici* 16	3.17 ± 0.08 (1.83 × 10^−6^)	3.09 ± 0.09 (1.91 × 10^−8^)	1.93 ± 0.07 (3.07 × 10^−9^)	2.57 ± 0.08 (2.43 × 10^−10^)
*L. rhamnosus* 0997	3.00 ± 0.04 (2.00 × 10^−6^)	2.77 ± 0.10 (2.23 × 10^−8^)	2.47 ± 0.11 (2.53 × 10^−9^)	2.31 ± 0.04 (2.69 × 10^−10^)
**Initial concentration of AA 50 [µg/mL]**				
**Strain**	**Remaining concentration of AA [µg/mL]** **(AA bound per 1 cell)**			
	**10^6^**	**10^8^**	**10^9^**	**10^10^**
*L. citreum* 50	30.38 ± 0.31 (19.62 × 10^−6^)	30.85 ± 0.55 (19.15 × 10^−8^)	23.54 ± 0.29 (26.46 × 10^−9^)	22.26 ± 0.24 (27.74 × 10^−10^)
*L. lactis* 3	31.67 ± 0.33 (18.77 × 10^−6^)	31.04 ± 0.42 (18.96 × 10^−8^)	23.62 ± 0.35 (26.38 × 10^−9^)	23.78 ± 0.64 (26.22 × 10^−10^)
*K. lactis* D2	27.12 ± 0.23 (22.88 × 10^−6^)	29.10 ± 0.39 (20.90 × 10^−8^)	20.24 ± 0.47 (29.76 × 10^−9^)	11.80 ± 0.34 (38.20 × 10^−10^)
*K. barnettii* D8	29.85 ± 0.43 (20.15 × 10^−6^)	31.68 ± 0.23 (18.32 × 10^−8^)	26.38 ± 0.30 (23.62 × 10^−9^)	27.43 ± 0.51 (22.57 × 10^−10^)

**Table 4:** The ability of heat-treated (100 °C) LAB and yeast to bind AA to the cell wall after 24 h of incubation at 37 °C. The pH was 7.0, and the cell density was 10^9^ CFU/mL. Results are presented as the mean from two measurements ± standard deviation (SD).

Initial Concentration of AA 5 µg/mL	
Strain	Remaining Concentration of AA [µg/mL]
*L. plantarum* 52	2.78 ± 0.04
*P. acidilactici* 16	2.77 ± 0.01
*L. rhamnosus* 0997	2.89 ± 0.07
**Initial concentration of AA 50 µg/mL**	
**Strain**	**Remaining concentration of AA [µg/mL]**
*L. citreum* 50	28.11 ± 0.37
*L. lactis* 3	27.39 ± 0.31
*K. lactis* D2	27.39 ± 0.44
*K. barnettii* D8	28.23 ± 0.51

**Table 5:** The amount of AA bound to intracellular and membrane extracts after 24 h of incubation at 37 °C with selected LAB and yeast strains. Results are presented as the mean from two measurements ± standard deviation (SD).

Initial Concentration of AA 5 [µg/mL]		
Strain	Remaining Concentration of AA [µg/mL]	
	Intracellular Extracts	Membrane Extracts
*L. plantarum* 52	0.42 ± 0.03	0.49 ± 0.02
*P. acidilactici* 16	0.47 ± 0.01	0.54 ± 0.00
*L. rhamnosus* 0997	0.62 ± 0.02	0.61 ± 0.01
**Initial concentration of AA 50 [µg/mL]**		
**Strain**	**Remaining concentration of AA [µg/mL]**	
	**Intracellular extracts**	**Membrane extracts**
*L. citreum* 50	0.87 ± 0.03	0.94 ± 0.09
*L. lactis* 3	1.23 ± 0.11	1.51 ± 0.04
*K. lactis* D2	0.75 ± 0.05	0.91 ± 0.03
*K. barnettii* D8	0.80 ± 0.02	0.94 ± 0.01

## Data Availability

The data presented in this study are available in this article and are available from the corresponding author upon reasonable request.

## Supplementary Materials

The following supporting information can be downloaded at: https://www.mdpi.com/article/10.3390/molecules29204922/s1, Table S1: Lactic acid bacteria strains used in the study with described isolation source.; Table S2: Yeast strains used in the study with described isolation source.; Table S3: Effect of acrylamide (5, 10, 50 μg/mL) on the growth of lactic acid bacteria during 24 h of incubation based on the spectrophotometric method. Each data point represents the mean from eight individual wells. The evaluation was conducted with two or three independent experiments. The differences from the unexposed control were significant at * *p* ≤ 0.0332, and ** *p* ≤ 0.0021.; Table S4: Effect of acrylamide (5, 10, 50 μg/mL) on the growth of yeast during 24 h of incubation based on the spectrophotometric method. Each data point represents the mean from eight individual wells. The evaluation was conducted with two or three independent experiments. The differences from the unexposed control were significant at * *p* ≤ 0.0332, ** *p* ≤ 0.0021, and **** *p* ≤ 0.0001.; Table S5: The ability of LAB cells to bind AA to the cell wall after 24 h of incubation at 37 °C. The pH was 7.0, and the cell density was 10^9^ CFU/mL. Results are presented as the mean from two measurements ± standard deviation (SD). Underlined strains with the defined AA dosage were chosen for the further experiments under various conditions.; Table S6: The ability of yeast cells to bind AA to the cell wall after 24 h of incubation at 37 °C. The pH was 7.0, and the cell density was 10^9^ CFU/mL. Results are presented as mean from two measurements ± standard deviation (SD). Underlined strains with the defined AA dosage were chosen for the further experiments under various conditions.
