# Supplementary material for: Detoxification of Acrylamide by Potentially Probiotic Strains of Lactic Acid Bacteria and Yeast
Source: Molecules. 2024 Oct 17;29(20):4922. doi: 10.3390/molecules29204922 (PMC11510321; doi:10.3390/molecules29204922)
Supplement: Supplementary file 1 [file molecules-29-04922-s001.zip › Table S1 - final.pdf]

Detoxification of Acrylamide by Potentially Probiotic Strains of Lactic Acid Bacteria and Yeast

Agnieszka Maher [1,*], Karolina Miśkiewicz [2], Justyna Rosicka-Kaczmarek [2], and Adriana Nowak [1]

[1] Department of Environmental Biotechnology, Faculty of Biotechnology and Food Sciences, Lodz University of Technology, Wolczanska 171/173, 90-530 Lodz, Poland; agnieszka.maher@dokt.p.lodz.pl; adriana.nowak@p.lodz.pl

[2] Institute of Food Technology and Analysis, Faculty of Biotechnology and Food Sciences, Lodz University of Technology, Stefanowskiego 2/22, 90-537 Lodz, Poland; karolina.miskiewicz@p.lodz.pl; justyna.rosicka-kaczmarek@p.lodz.pl

* Correspondence: agnieszka.maher@dokt.p.lodz.pl

Supplementary Materials

Table S1. Lactic acid bacteria strains used in the study with described isolation source.

LAB strain	Isolation source
Levilactobacillus brevis 1	Sourdough
Levilactobacillus brevis 2	
Lactococcus lactis 3	
Lactiplantibacillus plantarum 4	
Levilactobacillus brevis 5	
Levilactobacillus brevis 6	
Pediococcus acidilactici 7	
Pediococcus acidilactici 8	
Leuconostoc mesenteroides 10	
Lactiplantibacillus plantarum 14	
Pediococcus acidilactici 16	
Lactiplantibacillus plantarum 17	
Lactiplantibacillus plantarum 19	
Levilactobacillus brevis 20	
Pediococcus pentosaceus 21	
Levilactobacillus brevis 22	
Leuconostoc lactis 25	
Lactiplantibacillus plantarum 26	
Lactiplantibacillus plantarum 28	
Limosilactobacillus fermentum 30	
Levilactobacillus brevis 36	
Secundilactobacillus collinoides 38	
Lactiplantibacillus plantarum 39	
Leuconostoc citreum 40	
Lactiplantibacillus plantarum 41	
Lactiplantibacillus pentosus 42	
Lactiplantibacillus plantarum 43	
Leuconostoc citreum 44	
Levilactobacillus brevis 45	

Levilactobacillus brevis 46	
Lactiplantibacillus plantarum 47	
Levilactobacillus brevis 48	
Lactococcus raffinolactis 49	
Leuconostoc citreum 50	
Lactiplantibacillus pentosus 51	
Lactiplantibacillus plantarum 52	
Lactiplantibacillus plantarum 53	
Lactiplantibacillus plantarum 56	
Ligilactobacillus salivarius 9AN	Chicken pile with litter
Loigolactobacillus coryniformis 10AN	Sauerkraut
Lacticaseibacillus casei 12AN	Human faeces
Limosilactobacillus fermentum 57A	No data
Lentilactobacillus farraginis T6	Jerusalem artichoke
Levilactobacillus brevis OK-C	Sour cucumbers
Lacticaseibacillus paracasei OK-D	
Lactiplantibacillus plantarum OK-EK	
Levilactobacillus brevis OK-EJ	
Pediococcus parvulus OK-S	
Secundilactobacillus similis 04/2	
Pediococcus acidilactici 7/1	Flowers of *Papaver rhoeas*
Pediococcus pentosaceus 14/1	Flowers of *Lavandula augustifolia*
Lactiplantibacillus plantarum 21/1	Fresh fermented honey from the beekeeper
Pediococcus acidilactici 25/1	Nectar-heather honey
Lacticaseibacillus rhamnosus GG	Human faeces
Lactobacillus acidophilus ŁOCK 0839	No data
Amylolactobacillus amylophilus ŁOCK 0843	
Lactiplantibacillus plantarum ŁOCK 0981	Sour cucumbers
Levilactobacillus brevis ŁOCK 0983	
Lactobacillus delbrueckii ŁOCK 0987	Infant faces
Leuconostoc mesenteroides ŁOCK 0994	Curdled cow milk
Lacticaseibacillus rhamnosus ŁOCK 0997	Infant faces
