# Supplementary material for: Detoxification of Acrylamide by Potentially Probiotic Strains of Lactic Acid Bacteria and Yeast
Source: Molecules. 2024 Oct 17;29(20):4922. doi: 10.3390/molecules29204922 (PMC11510321; doi:10.3390/molecules29204922)
Supplement: Supplementary file 1 [file molecules-29-04922-s001.zip › Table S2 - final.pdf]

Table S2. Yeast strains used in the study with described isolation source.

**Yeast strain**	**Isolation source**
Saccharomyces cerevisiae D1	Cider
Kluyveromyces lactis ŁOCK 0028 D2	No data
Metschnikowia pulcherrima NCYC 747 D3	
Saccharomyces cerevisiae D4	Rye flour
Saccharomyces cerevisiae D5	BIO STAR company
Pichia fermentans D6	Sour cucumber
Wickerhamomyces anomalus D7	Wheat flour
Kazachstania barnettii D8	Sour beet root
Kluyveromyces marxianus ŁOCK 0024 D9	No data
Hanseniaspora uvarum D10	Wild grape 1
Hanseniaspora uvarum D11	Wild grape 2
Saccharomyces cerevisiae D12	Multivitamin drink
Wickerhamomyces anomalus D13	Sour rye soup
* (-) D14	Blackberry fruit
Metschnikowia pulcherrima CCY-2-145 D15	No data

* identification in progress.
