# Supplementary material for: Detoxification of Acrylamide by Potentially Probiotic Strains of Lactic Acid Bacteria and Yeast
Source: Molecules. 2024 Oct 17;29(20):4922. doi: 10.3390/molecules29204922 (PMC11510321; doi:10.3390/molecules29204922)
Supplement: Supplementary file 1 [file molecules-29-04922-s001.zip › Table S3 - final.pdf]

**Table S3.** Effect of acrylamide (5, 10, 50 μg/ml) on the growth of lactic acid bacteria during 24 h of incubation based on the spectrophotometric method. Each data point represents the mean from eight individual wells. The evaluation was conducted with two or three independent experiments. The differences from the unexposed control were significant at * $p \leq 0.0332$ and ** $p \leq 0.0021$.

| LAB strain | 5 μg/ml AA [% control] | 10 μg/ml AA [% control] | 50 μg/ml AA [% control] |
|---|---|---|---|
| *L. brevis* 1 | 100.07±1.01 | 100.20±2.77 | 99.11±3.18 |
| *L. brevis* 2 | 100.26±2.58 | 104.08±4.29 | 105.06±3.23 |
| *L. lactis* 3 | 100.83±4.97 | 105.44±2.56 | 100.63±3.55 |
| *L. plantarum* 4 | 99.03±1.07 | 98.95±2.04 | 100.00±1.73 |
| *L. brevis* 5 | 99.75±1.46 | 100.21±0.89 | 100.26±2.58 |
| *L. brevis* 6 | 100.47±0.88 | 101.40±0.76 | 99.62±0.67 |
| *P. acidilactici* 7 | 100.28±5.83 | 104.18±3.84 | 101.31±4.35 |
| *P. acidilactici* 8 | 94.07±20.77 | 83.16±1.85 | 92.09±5.10 |
| *L. mesenteroides* 10 | 101.53±1.94 | 101.52±2.17 | 101.60±1.41 |
| *L. plantarum* 14 | 109.12±2.86 | 101.92±5.49 | 103.85±8.04 |
| *P. acidilactici* 16 | 100.27±0.81 | 100.95±0.86 | 100.37±1.07 |
| *L. plantarum* 17 | 95.38±1.77 | 97.68±3.24 | 92.73±3.70 |
| *L. plantarum* 19 | 102.17±5.74 | 102.28±3.90 | 99.09±4.75 |
| *L. brevis* 20 | 99.87±1.05 | 99.34±1.18 | 99.40±1.07 |
| *P. pentosaceus* 21 | 101.87±1.11 | 90.79±20.06 | 95.22±12.13 |
| *L. brevis* 22 | 101.34±2.88 | 101.01±3.71 | 100.08±6.82 |
| *L. lactis* 25 | 99.02±3.20 | 98.94±4.38 | 99.48±3.02 |
| *L. plantarum* 26 | 97.59±10.85 | 93.26±3.35 | 99.88±4.88 |
| *L. plantarum* 28 | 95.90±2.75 | 107.28±6.29 | 101.25±11.49 |
| *L. fermentum* 30 | 100.11±0.67 | 100.86±1.17 | 100.70±1.93 |
| *L. brevis* 36 | 103.04±4.30 | 104.49±1.89 | 101.80±1.00 |
| *S. collinoides* 38 | 93.33±3.62 | 82.67±4.92 ** | 87.02±6.50 * |
| *L. plantarum* 39 | 106.56±3.64 | 102.19±2.51 | 100.37±7.75 |
| *L. citreum* 40 | 89.95±15.32 | 87.32±2.12 | 89.18±1.08 |
| *L. plantarum* 41 | 96.47±13.13 | 97.90±7.65 | 99.96±6.04 |
| *L. pentosus* 42 | 106.56±4.60 | 110.19±4.82 | 110.76±4.27 |
| *L. plantarum* 43 | 100.56±3.05 | 100.47±2.02 | 100.26±2.92 |
| *L. citreum* 44 | 105.16±10.48 | 102.32±2.67 | 98.93±4.87 |
| *L. brevis* 45 | 98.85±1.22 | 91.32±12.29 | 89.00±8.98 |
| *L. brevis* 46 | 101.00±3.35 | 98.91±4.05 | 98.81±6.38 |
| *L. plantarum* 47 | 99.53±1.56 | 100.01±2.32 | 99.21±2.17 |
| *L. brevis* 48 | 103.12±1.21 | 99.00±3.49 | 99.77±3.84 |
| *L. raffinolactis* 49 | 92.18±2.48 | 90.34±8.14 | 91.21±1.62 |
| *L. citreum* 50 | 98.28±2.01 | 99.11±0.62 | 97.50±2.24 |
| *L. pentosus* 51 | 111.27±14.97 | 90.56±4.20 | 96.36±7.34 |
| *L. plantarum* 52 | 89.25±1.45 | 72.71±30.86 * | 91.65±6.07 |
| *L. plantarum* 53 | 99.85±1.67 | 99.90±1.78 | 100.51±1.90 |
| *L. plantarum* 56 | 100.67±3.06 | 100.06±1.15 | 99.61±2.20 |
| *L. salivarius* 9AN | 100.13±1.83 | 97.86±0.89 | 98.12±0.74 |
| *L. coryniformis* 10AN | 100.03±0.99 | 100.21±0.72 | 100.71±0.97 |
| *L. casei* 12AN | 101.03±1.05 | 99.79±0.56 | 97.28±0.97 |
| *L. fermentum* 57A | 100.19±1.98 | 100.16±1.71 | 100.50±2.13 |
| *L. farraginis* T6 | 102.48±1.13 | 102.48±1.61 | 102.94±1.37 |
| *L. brevis* OK-C | 100.50±1.03 | 100.62±0.72 | 100.92±1.41 |
| *L. paracasei* OK-D | 99.86±0.91 | 100.22±0.86 | 99.34±0.83 |
| *L. plantarum* OK-EK | 100.47±1.05 | 100.53±1.01 | 100.38±0.42 |
| *L. brevis* OK-EJ | 100.89±1.43 | 100.11±1.31 | 96.77±0.46 |

| | | | |
|---|---|---|---|
| *P. parvulus* OK-S | 100.19±1.20 | 100.58±2.01 | 100.03±0.68 |
| *S. similis* 04/2 | 99.91±0.78 | 100.77±1.16 | 100.28±0.68 |
| *P. acidilactici* 7/1 | 99.99±1.02 | 100.04±0.54 | 99.61±0.84 |
| *P. pentosaceus* 14/1 | 102.74±0.77 | 101.70±2.21 | 101.93±1.90 |
| *L. plantarum* 21/1 | 101.13±1.91 | 101.44±2.23 | 98.89±1.31 |
| *P. acidilactici* 25/1 | 101.73±1.15 | 101.39±0.92 | 98.84±0.98 |
| *L. rhamnosus* GG | 99.92±0.95 | 99.43±1.18 | 99.63±1.03 |
| *L. acidophilus* 0839 | 100.02±0.78 | 97.54±1.04 | 96.12±1.09 |
| *A. amylophilus* 0843 | 101.48±1.45 | 99.01±0.81 | 98.16±0.75 |
| *L. plantarum* 0981 | 98.87±0.88 | 99.45±0.96 | 98.86±2.24 |
| *L. brevis* 0983 | 100.51±0.94 | 101.21±0.85 * | 100.05±1.34 |
| *L. delbrueckii* 0987 | 100.33±1.73 | 96.16±1.38 | 94.63±1.15 * |
| *L. mesenteroides* 0994 | 99.48±0.58 | 97.51±1.44 | 97.54±0.47 |
| *L. rhamnosus* 0997 | 110.39±3.62 | 109.77±1.58 | 110.95±2.52 |
| **Average** | 100.28±3.88 | 98.87±6.09 | 98.95±4.14 |
