# Supplementary material for: Detoxification of Acrylamide by Potentially Probiotic Strains of Lactic Acid Bacteria and Yeast
Source: Molecules. 2024 Oct 17;29(20):4922. doi: 10.3390/molecules29204922 (PMC11510321; doi:10.3390/molecules29204922)
Supplement: Supplementary file 1 [file molecules-29-04922-s001.zip › Table S4 - final.pdf]

Table S4. Effect of acrylamide (5, 10, 50 μg/ml) on the growth of yeast during 24 h of incubation based on the spectrophotometric method. Each data point represents the mean from eight individual wells. The evaluation was conducted with two or three independent experiments. The differences from the unexposed control were significant at * $p \leq 0.0332$, ** $p \leq 0.0021$, and **** $p \leq 0.0001$.

**Yeast strain**	**5 μg/ml AA [% control]**	**10 μg/ml AA [% control]**	**50 μg/ml AA [% control]**
S. cerevisiae D1	98.82±1.48	99.86±0.95	100.75±1.58
K. lactis D2	104.05±3.40	104.32±8.49	99.15±3.53
M. pulcherrima D3	98.32±6.65	99.42±2.54	98.00±2.30
S. cerevisiae D4	101.56±3.66	102.61±2.73	100.27±4.65
S. cerevisiae D5	98.39±4.40	98.08±3.58	126.67±4.31
P. fermentans D6	101.52±3.37	91.10±10.72	92.20±20.45
W. anomalus D7	102.42±2.85	100.35±4.74	97.15±1.61
K. barnettii D8	106.00±9.06	89.21±6.34 *	91.44±10.50
K. marxianus D9	104.48±6.14	103.06±1.79	108.38±5.45 *
H. uvarum D10	96.80±1.67	98.25±2.27	96.81±1.08
H. uvarum D11	102.50±1.73	102.03±1.28	100.15±2.20
S. cerevisiae D12	105.73±7.15	101.88±4.73	93.94±3.53
W. anomalus D13	98.13±5.76	96.47±6.29	90.26±3.85 ****
(-) D14	101.16±1.64	100.06±2.77	98.26±2.75
M. pulcherrima D15	107.30±6.56	85.73±6.64 **	95.18±5.35
**Average**	101.81±4.37	98.16±4.39	99.24±4.88
