# Supplementary material for: Detoxification of Acrylamide by Potentially Probiotic Strains of Lactic Acid Bacteria and Yeast
Source: Molecules. 2024 Oct 17;29(20):4922. doi: 10.3390/molecules29204922 (PMC11510321; doi:10.3390/molecules29204922)
Supplement: Supplementary file 1 [file molecules-29-04922-s001.zip › Table S5 - final.pdf]

Table S5. The ability of LAB cells to bind AA to the cell wall after 24 h of incubation at 37°C. The pH was 7.0, and the cell density was 10^9 CFU/ml. Results are presented as the mean from two measurements ± standard deviation (SD). Underlined strains with the defined AA dosage were chosen for the further experiments under various conditions.

Strain	Initial concentration of AA 5 μg/ml	Initial concentration of AA 50 μg/ml
	Remaining concentration of AA [μg/ml]	
L. brevis 2	3.85±0.07	38.20±0.66
L. lactis 3	3.06±0.06	23.62±0.21
L. brevis 5	3.91±0.09	37.28±0.90
L. brevis 6	4.26±0.04	39.09±0.34
P. acidilactici 7	3.80±0.03	35.67±0.65
P. acidilactici 8	2.54±0.06	31.58±0.32
L. mesenteroides 10	3.56±0.03	28.01±0.53
L. plantarum 14	3.26±0.06	44.30±0.78
P. acidilactici 16	1.93±0.10	25.77±0.82
L. plantarum 17	4.17±0.05	36.57±0.48
L. plantarum 19	3.81±0.04	35.63±0.37
P. pentosaceus 21	2.64±0.03	29.90±0.63
L. brevis 22	2.61±0.08	27.29±0.94
L. lactis 25	3.00±0.07	23.56±0.56
L. plantarum 26	3.86±0.05	38.40±0.72
L. fermentum 30	2.58±0.06	29.34±0.84
S. collinoides 38	2.49±0.08	23.19±0.28
L. plantarum 39	3.34±0.05	39.36±0.42
L. citreum 40	3.70±0.09	31.22±0.85
L. plantarum 41	3.89±0.04	40.52±0.27
L. pentosus 42	2.55±0.04	31.86±0.51
L. plantarum 43	4.08±0.03	36.91±0.80
L. citreum 44	3.91±0.05	41.99±0.41
L. brevis 45	2.24±0.06	27.10±0.73
L. brevis 46	3.72±0.04	27.78±0.42
L. plantarum 47	3.83±0.09	39.12±0.89
L. raffinolactis 49	4.06±0.10	38.19±0.41
L. citreum 50	2.63±0.08	23.54±0.39
L. pentosus 51	2.45±0.02	27.97±0.63
L. plantarum 52	2.23±0.03	27.01±0.84
L. plantarum 53	4.13±0.04	36.18±0.38
L. plantarum 56	2.96±0.03	28.07±0.47
L. casei 12AN	4.62±0.06	37.51±0.49
L. paracasei OK-D	2.42±0.08	29.74±0.52
P. parvulus OK-S	3.94±0.04	45.08±0.77
L. mesenteroides 0994	3.02±0.02	23.87±0.84
L. rhamnosus 0997	2.47±0.04	28.68±0.34
