# Supplementary material for: Detoxification of Acrylamide by Potentially Probiotic Strains of Lactic Acid Bacteria and Yeast
Source: Molecules. 2024 Oct 17;29(20):4922. doi: 10.3390/molecules29204922 (PMC11510321; doi:10.3390/molecules29204922)
Supplement: Supplementary file 1 [file molecules-29-04922-s001.zip › Table S6 - final.pdf]

Table S6. The ability of yeast cells to bind AA to the cell wall after 24 h of incubation at 37°C. The pH was 7.0, and the cell density was 10^9 CFU/ml. Results are presented as the mean from two measurements ± standard deviation (SD). Underlined strains with the defined AA dosage were chosen for the further experiments under various conditions.

**Strain**	**Initial concentration of AA 5 µg/ml**	**Initial concentration of AA 50 µg/ml**
	**Remaining concentration of AA [µg/ml]**	
S. cerevisiae D1	3.24±0.02	30.93±0.44
K. lactis D2	3.47±0.06	20.24±0.15
M. pulcherrima D3	2.92±0.03	33.75±0.37
S. cerevisiae D4	3.39±0.03	38.73±0.98
S. cerevisiae D5	3.55±0.05	36.60±0.88
P. fermentans D6	3.38±0.09	37.20±0.54
W. anomalus D7	2.90±0.07	26.01±0.33
K. barnettii D8	4.03±0.01	26.38±0.41
K. marxianus D9	2.71±0.05	26.46±0.76
H. uvarum D10	3.76±0.03	36.84±0.81
S. cerevisiae D12	2.97±0.07	31.22±0.14
W. anomalus D13	3.72±0.09	35.37±0.36
(-) D14	2.89±0.02	25.81±0.29
